# Impact of stagnation and sampling volume on water microbial quality monitoring in large buildings

**DOI:** 10.1371/journal.pone.0199429

**Published:** 2018-06-21

**Authors:** Emilie Bédard, Céline Laferrière, Eric Déziel, Michèle Prévost

**Affiliations:** 1 Department of Civil Engineering, Polytechnique Montréal, Montréal, QC, Canada; 2 INRS-Institut Armand-Frappier, Laval, QC, Canada; 3 Department of Microbiology and Immunology (Infection control), CHU Ste-Justine, Université de Montréal, Montréal, QC, Canada; Nanjing University, CHINA

## Abstract

Microbial drinking water quality can be altered in large buildings, especially after stagnation. In this study, bacterial profiles were generated according to the stagnation time and the volume of water collected at the tap. Successive volumes of cold and hot water were sampled after controlled stagnation periods. Bacterial profiles revealed an important decline (> 2 log) in culturable cells in the first 500 mL sampled from the hot and cold water systems, with a steep decline in the first 15 mL. The strong exponential correlation (R^2^ ≥ 0.97) between the culturable cell counts in water and the pipe surface-to-volume ratio suggests the biofilm as the main contributor to the rapid increase in suspended culturable cells measured after a short stagnation of one-hour. Results evidence the contribution of the high surface-to-volume ratio at the point of use and the impact of short stagnation times on the increased bacterial load observed. Simple faucets with minimal internal surface area should be preferred to minimize surface area. Sampling protocol, including sampling volume and prior stagnation, was also shown to impact the resulting culturable cell concentration by more than 1000-fold. Sampling a smaller volume on first draw after stagnation will help maximize recovery of bacteria.

## Introduction

Drinking water microbial quality is regulated and monitored prior to, and throughout, municipal distribution systems, ensuring quality water is delivered to the consumer’s premise plumbing [1] and regrowth of microorganisms is minimized. However, monitoring of microbial quality after stagnation in premise plumbing is generally not performed, despite the significant quality alterations that can occur, especially within large buildings [2–4]. Biofilm formation, periodical stagnation, high surface-to-volume ratios, favorable temperatures and pipe materials are factors that can promote bacterial growth in premise plumbing [5, 6].

In healthcare building premise plumbing, culturable bacteria levels are used as an indicator of the risk for opportunistic pathogen infections, as illustrated by infection prevention guidelines for *Legionella pneumophila* and *Pseudomonas aeruginosa* [7–9]. In France, an increase of culturable cells by more than 1 log between the cold water infeed and the point-of-use is considered an abnormal variation and indicative of regrowth within the system [7]. Heterotrophic plate counts (HPC) were found to be a reliable indicator to monitor regrowth in the drinking water environment, together with more specific *Aeromonas* and *Mycobacterium* measurements [10]. Culturable cell counts are valuable to identify under which conditions the abundance of microorganisms can increase in premise plumbing water. However, culture-based methods do not provide indications on the presence of viable but non culturable (VBNC) cells in full scale distribution systems and premise plumbing [11, 12]. VBNC cells may present a health risk as they retain and are able to regain virulence, together with their culturability, under suitable conditions [13].

Previous studies reported microbial amplification in distal points of large building premise plumbing, with 5- to 30-fold increases observed in HPC between the plumbing system and the points-of-use [5, 14, 15]. The distal points refer to points of use such as taps and showers, and their connection to the principal water distribution system. Comparisons are typically done between a first flush sample of 1 liter at a point-of-use and a sample from the principal water system taken after flushing (1 to 5 minutes). Culturable and viable bacterial profiling in full scale buildings show a decrease from the first liter and then progressively with flushing in the cold and hot water from premise systems [2, 16, 17]. These observations raise important questions with regards to the choice of sampling strategy, especially the use of flushing and the sampling volume, which could greatly affect the results obtained.

Previous investigations on the role of water stagnation in the distal microbial load increase have revealed the importance of overnight inactivity [2, 16, 18]. The hypothesis is that amplification is caused by a combination of bacterial growth, bacterial cell detachment and sloughing from the biofilm during stagnation and flow. The risk of exposure to bacteria can be reduced by implementing a practice of flushing taps for 1 to 5 min before use, a procedure recommended in areas inactive for prolonged periods of times [9]. However, extended flushing of all faucets after overnight stagnation can be time-consuming, especially in large buildings with multiple points-of-use such as in healthcare facilities. In addition, the impact of shorter stagnation periods frequently occurring throughout the day is poorly documented. There are very few reports of the impact of stagnation on the microbial profiles in the first liters of hot water [19].

The main objective of the present study was to establish the bacterial load profile in cold and hot water systems according to the stagnation time and the volume of water collected at the point-of-use, in order to define an optimum sampling protocol and better interpret sampling results. The results will also reveal where are located the bacteria in the premise plumbing and if short stagnation periods encountered throughout the day impact bacterial loads in the water.

## Materials and methods

### Description of the study site

The study was performed in a ten-story 450 bed children’s hospital in Canada (45°30'10"N, 73°37'26"W), fed by chlorinated surface filtered drinking water. The cold water system was sampled in July 2012 with an incoming municipal water mean temperature of 26.2°C, a measured residual chlorine of 0.4 mg Cl_2_/L, an average of 5x10^-3^ CFU/mL and 7x10^3^ viable cells/mL. The mean water temperature exiting the heater and feeding into the hot water system was 61.6°C and the residual chlorine concentration was below 0.1 mg Cl_2_/L. The hot water system was sampled between November and December 2012.

### Sampling protocol

**Fig 1:**
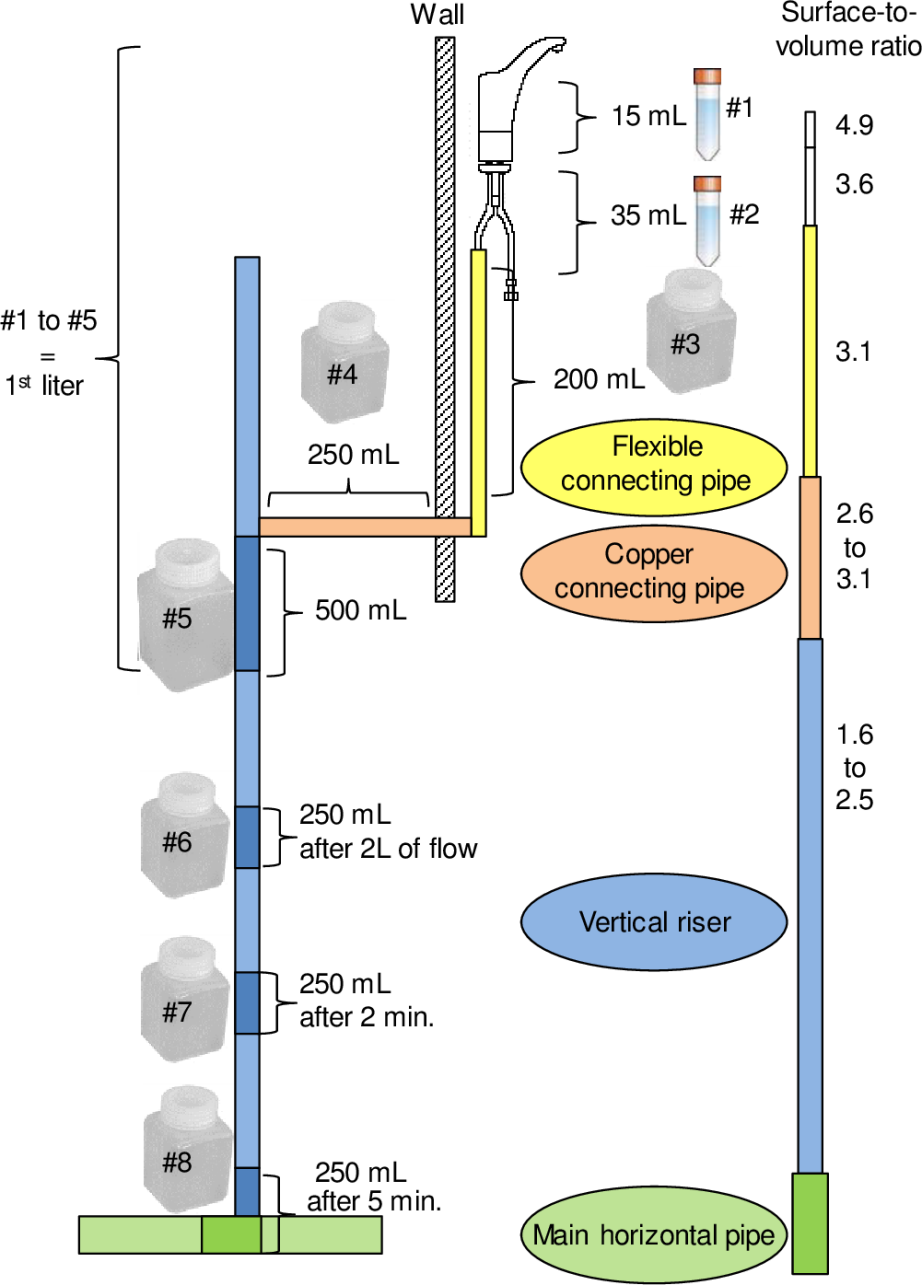
Sampling sequence illustrating the origin of the water within the premise plumbing, and estimated surface-to-volume ratio for each section.

The sampling was conducted separately for cold and hot water systems, on two designated manual taps that were dedicated exclusively to the study throughout its duration, without additional water usage. Hot and cold water systems were sampled in separate events to ensure controlled stagnation of the first liter. Each water system was sampled twice at each tap for each stagnation time. An initial flush of 5-min was conducted on the tap prior to the start of stagnation. Sampling was performed immediately upon opening the water, without prior sterilization of the tap, after 1, 24, 48, 72, 120 and 240 hours of controlled stagnation, for a total of 6 sampling events for each water system (hot and cold). For each sampling event at a tap, successive separate volumes composing the first liter were sampled in sterile 50 mL tubes or propylene bottles: 1^st^ volume of 15 mL, 2^nd^ volume of 35 mL, 3^rd^ volume of 200 mL, 4^th^ volume of 250 mL, 5^th^ volume of 500 mL (Fig 1). Two additional samples of 250 mL were collected after 2 L and 5 L of flow. The last sample was collected after 5 min of flow, corresponding to an average volume of 9.1 L, at an average flow rate of 0.9 L/min. A low flow rate was set to facilitate the collection of small volumes within the first liter. The flow was not changed during sampling to avoid change of flow regime. Sampling volumes and flush times were selected as representative of different sections within the premise plumbing (Fig 1), based on the studied system architecture: the first volume (15 mL) corresponds to the water within the body of the faucet; the second volume (35 mL) corresponds to the water that stagnated within the tap connection; the third volume (200 mL) is indicative of the water that stagnated in the flexible connecting pipes between the faucet and the wall; the fourth volume (250 mL) is associated to the water in the copper pipe, connecting to the vertical riser; the fifth volume (500 mL) and samples collected after 2 L and 5 L of flow represent water from the vertical riser. The last sample was collected after 5 min of flow and is representative of the main horizontal pipe. Based on the system architecture, it was estimated that the first 500 mL only were subject to controlled stagnation, as this section of the plumbing is specific to the tap. The other volumes were representatives of pipes that fed water to multiple outlets and didn’t experience full stagnation. The protocol was submitted and authorized by the head of the hospital department of microbiology and immunology and the director of technical services.

Full dechlorination of samples was achieved through the addition of sodium thiosulfate in the sampling container (final concentration 1.1 mg/L). The concentration of thiosulfate was optimized to ensure full dechlorination and yet minimize interferences with epifluorescence-based methods. For each sampled volume, HPCs, direct viable and total bacterial cell counts were assessed as described in the microbiological analysis section. Temperature, pH and residual chlorine were measured immediately following the first liter for each sampling event. Residual chlorine concentrations were measured with a Pocket Colorimeter II (HACH, USA). For hot water, temperature was also measured after the last sampled volume (5-min flush).

### Microbiological analysis

All samples were maintained at 4°C during storage and transportation, and analyzed within 24 hours from sampling. Heterotrophic plate counts (HPCs) were performed on R2A agar at 22°C, after 7 days of incubation according to method 9215-D [20]. Viable and total cell counts were determined by fluorescence microscopy following staining with the LIVE/DEAD BacLight Bacterial Viability Kit (Molecular Probes, Eugene, USA) [21]. This kit differentiates intact (viable) from damaged cells using membrane integrity criteria. Briefly, 1 mL of sample or dilution in 0.85% sterile saline solution was mixed with 3 μl of stain (propidium iodide and SYTO9), incubated in the dark for 15 min and filtered on a black 0.2 μm pore diameter, 25 mm diameter polycarbonate filter (Millipore, Bedford, USA). Enumeration was done with an epifluorescence microscope (Olympus) at 1000-fold magnification, on ten fields of view. Total bacterial cells are defined as the sum of intact (green) and damaged (red) cells.

## Results & discussion

In this study, water quality profiles were systematically performed at two taps, in cold and hot water systems from a large building. Variable controlled stagnation time periods were induced to understand the impact on water microbial quality for both cold and hot water systems. As described in Fig 1, different volumes were sampled successively during flushing of the tap to understand the contribution of each plumbing section on water quality.

### Culturable, viable and total cell profiles observed in cold and hot water

Culturable, viable and total cell profiles were determined in cold and hot water after stagnation times between 1h and 10 days. The pH and the chlorine residual were measured over time at each tap and in each water system. pH ranged between 7.5 and 8.1 (mean 7.8) and the chlorine residual measured after the first liter was between 0.05 and 0.45 mg Cl_2_/L (mean 0.25 mg Cl_2_/L) in cold water, and <LD–0.07 mg Cl_2_/L (mean 0.02 mg/L) in hot water. These variations in pH and chlorine residuals are within previously reported range in water samples collected from large building premise plumbing [22, 23]. Water temperature measured after 1 L of flow ranged between 24.1 and 27.7 °C (mean of 23.6°C) in cold water and between 24.6 and 47.5°C (mean of 35.5°C) in hot water. The large variation observed in the hot water temperature after one liter was attributed to tap location within the building. At the time of sampling, the hot water distribution system was poorly balanced hydraulically and hot water temperature at the tap varied depending on its location within the hospital [22]. As a result, the maximum hot water temperature for each of the two taps differed by close to 10°C (44.1 and 53.9°C).

**Fig 2:**
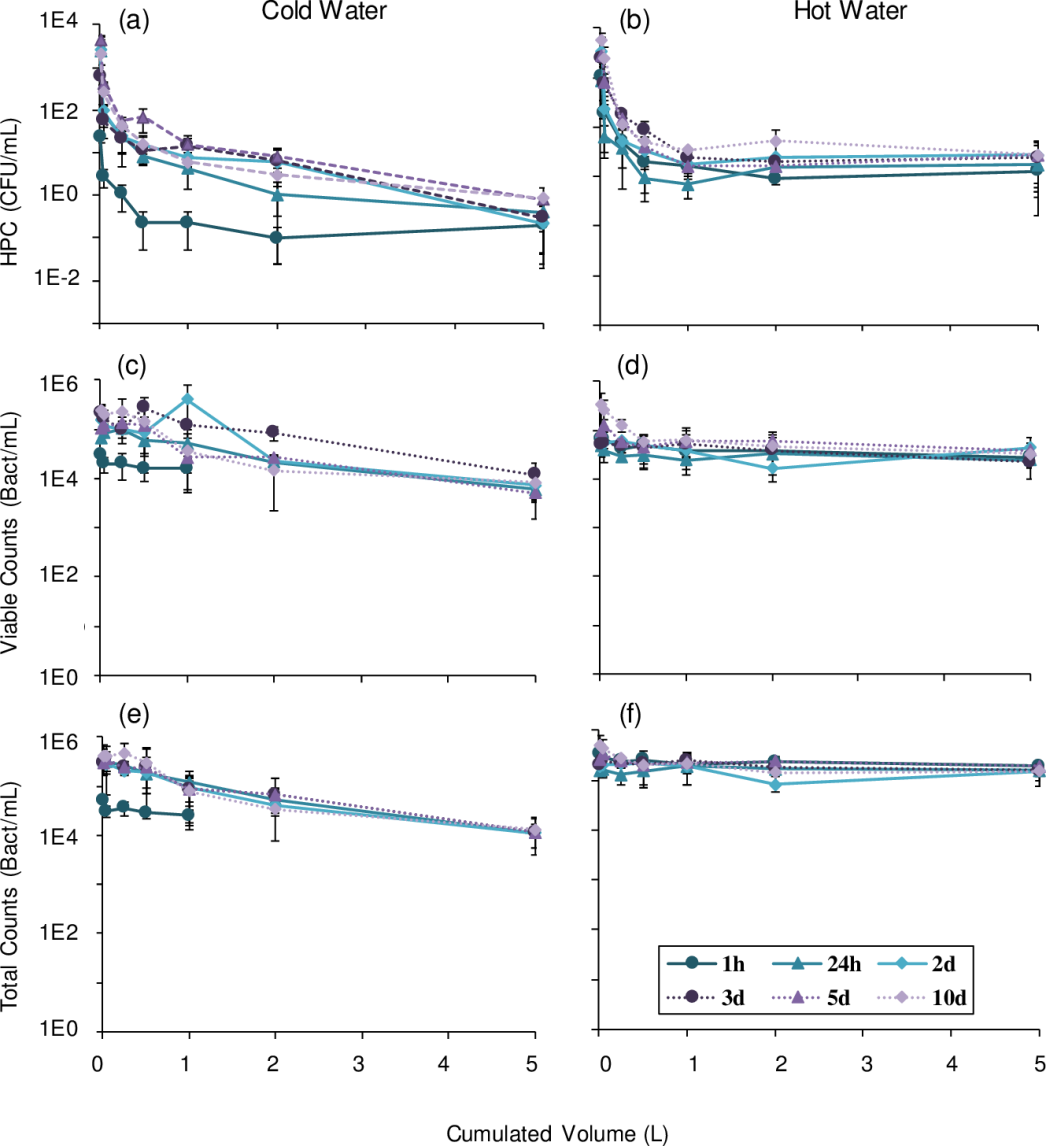
Mean HPC, viable and total cell count profiles from 2 taps for different stagnation times. Mean bacterial loads in water were evaluated in successive volumes of cold and hot water sampled from 2 taps. In cold water, (a) culturable, (c) viable and (e) total cells were plotted for stagnation time of 1 hour, 1, 2, 3, 5 and 10 days. Results measured in hot water are also shown for (b) culturable, (d) viable and (f) total cells.

Culturable bacteria profiles for all stagnation times were fitted with a power regression with a good correlation in cold water (R^2^ = 0.87–0.99) and hot water (R^2^ = 0.72–0.95). A rapid decline in culturable bacterial cell counts was observed in the first part of the profile in cold and hot water, between 0 and 0.5 L (Fig 2A and 2B). This volume corresponds to the pipe section attached upflow of the tap, and therefore experiencing full stagnation between samplings. In large buildings, stagnation of a given device will reflect on the volume of water within the faucet and immediate connecting piping to that device, while the rest of the system might circulate due to usage at other devices (cold and hot water) or forced recirculation (hot water).

The steepest slope decline is observed in the first 15 mL, corresponding to the water volume contained in the faucet, where the surface-to-volume ratio is maximum (Fig 1). The higher culturable cell counts per ml observed in this sample is likely associated with an increased presence of biofilm. The capacity for biofilm growth in piping and faucets is mainly determined by the surface available for colonization and the nature of the material [24–26]. Faucets contain a large number of internal parts (ball, cylinder or cartridge assembly) and seals in contact with water (S1 Fig). These assemblies result in recesses and crevices that add up to large projected surfaces, providing more attachment sites for biofilm development. In addition to the large surfaces present, wetted elements within faucets are generally made of various plastic and elastomeric materials that are favorable to biofilm growth [24, 27].

**Fig 3:**
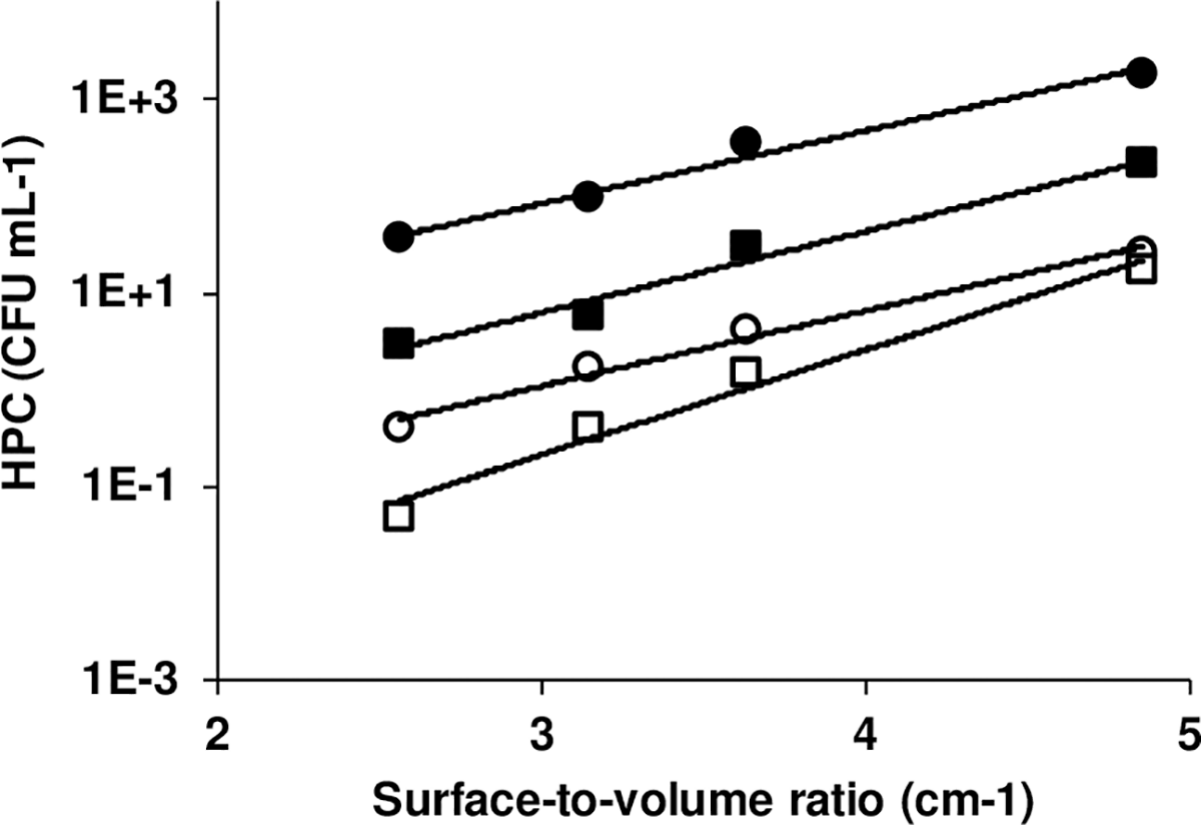
Culturable cell concentration after 1-hour stagnation as a function of surface-to-volume ratio in cold and hot water. An exponential correlation was observed between the HPC level and the surface-to-volume ratio corresponding to the section of the plumbing to which sampled water was associated. This was observed for both sampled taps in cold water (○: Tap 1, R^2^ = 0.99; □: Tap 2, R^2^ = 0.98) and hot water (●: Tap 1, R^2^ = 0.98; ■: Tap 2, R^2^ = 0.97).

To better understand the contribution of the biofilm to the high culturable cell counts observed at the faucet, surface-to-volume ratios were calculated for the various sections of the device plumbing corresponding to the first 500 mL of sampled water. A plot of culturable cell counts against the average surface-to-volume ratio reveals an excellent exponential correlation after one-hour stagnation (Fig 3, R^2^ ≥ 0.97) and similar trends for longer stagnation time (S2 Fig, R^2^ = 0.72–0.99). These results strongly suggest that release of biofilm bacteria is an important contributor to the increased culturable counts in water collected from the faucet and its connecting pipes. In healthcare facilities, simple faucets with minimal internal surface areas and simple or no aerators will reduce surfaces available for biofilm growth [28, 29]. The presence of flow restriction devices composed of complex structures with large surfaces of plastic materials are favorable to biofilm growth and can lead to outbreaks or colonization by opportunistic pathogens [14, 30].

Between 1 L and the 5-minute flush, the culturable cell count in cold water decreased from 7.8 CFU/mL (Fig 2A) to 0.39 CFU/mL (not shown), almost 2 log higher than levels in the incoming municipal water (5 x 10^−3^ CFU/mL). This suggests a contribution of the vertical and horizontal pipes to the increase observed within premise plumbing, despite a reduced surface-to-volume ratio and less stagnation due to water usage by other devices driving water circulation. In hot water, culturable cell counts leveled 0.3 to 1.3 log higher than in cold water (Fig 2A and 2B). In large buildings, hot water must be recirculated in order to maintain target temperatures throughout the system [31]. Thus, bacterial loads in vertical risers and main horizontal pipes are expected to be relatively uniform throughout the hot water distribution system, reflecting the growth conditions in the recirculating loops and the level of dilution provided by make-up water.

Differences in trends were noted for viable and total cell counts. The dynamic of HPCs was most notable in a very distal faucet volume, and somewhat controlled by flushing of the first 500 mL. However, viable and total cell count profiles did not display a similar decline in the first 500 mL (Fig 2C and 2E), suggesting that flushing a larger volume of water is required to reduce viable and total cell counts after stagnation in cold water. Profiles were comparable for all stagnation times in cold water, except after one-hour stagnation (Fig 2C and 2E). For stagnation times of 1 to 10 days, viable cell counts decreased by 1.6±0.3 log between the first 15 mL and 5 minutes of flushing, reaching levels found in the incoming cold water (7x10^3^ viable cells/mL). A similar decrease in cell concentration was also observed by Lautenschlager et al. between the first liter and 5 min flushed samples, after overnight stagnation of cold water [16]. Viable and total cell count profiles in hot water did not decrease with flushing (Fig 2D and 2F). Viable cell count profiles in hot water were stable and leveled to 2.7x10^4^ cells/mL (Fig 2D), 0.56 log above incoming cold water levels. Similar trends were observed for culturable cells and suggest a background contamination in the hot recirculating water system.

**Fig 4:**
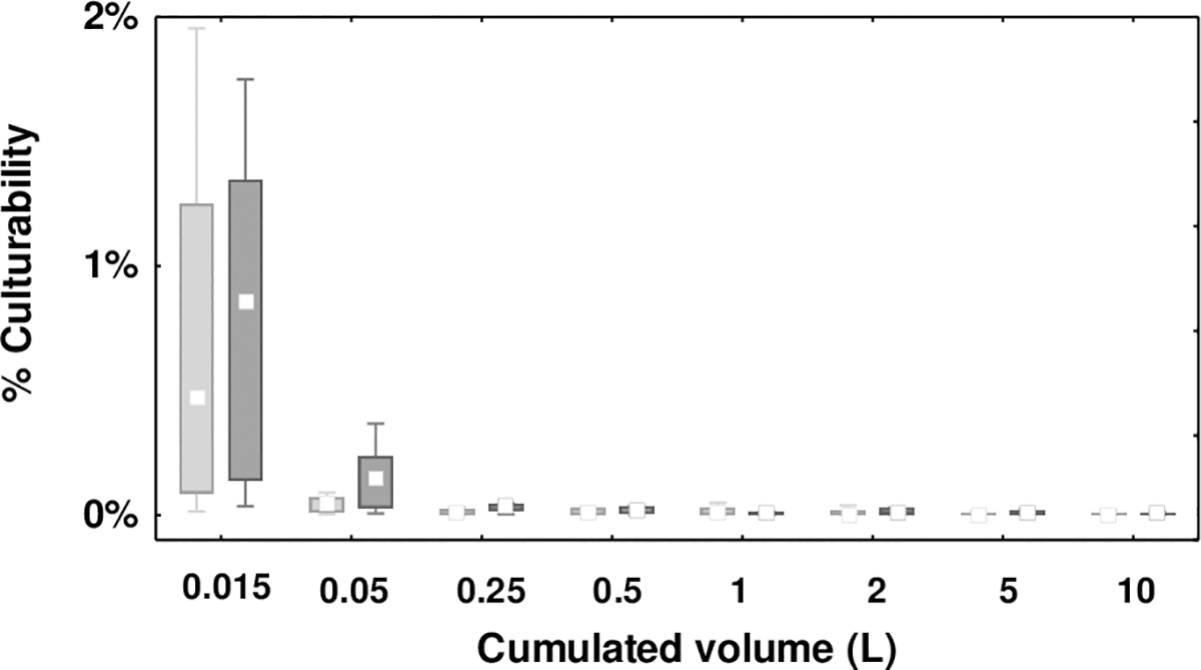
Percent culturability profiles in cold (light grey) and hot (dark gray) water systems measured at the faucet (n = 12). The box represents 25–75%, with the median and non-outlier range.

No correlation was observed between the viable and culturable cell counts in the first 15 mL sampled from the tap (R^2^ = 0.07, Panel a in S3 Fig), whereas a linear correlation was established in flushed samples (R^2^ = 0.69, Panel b in S3 Fig). The lack of correlation in the first 15 mL is associated to the large cell culturability variation observed within this first volume of water, which is also reflected on the percent of culturable cells measured in the different volumes sampled (Fig 4). High variability was observed in the first 15 mL whereas a low and stable percent culturability was measured in system water. Environmental stressors such as chlorine residual, pH, and copper concentrations in the stagnant volume at the tap can affect the recovery of culturable cells without affecting viable cell counts [32, 33]. During stagnation within the tap, water quality can deteriorate with temperature changes, residual disinfectant consumption, nutrient leaching materials and detachment from the biofilm. The increased correlation observed between culturable and viable cell counts after flushing reflects the more stable conditions encountered in the circulating water.

### Impact of short stagnation time and biofilm contribution to bacterial load in bulk water

Overnight or longer stagnation periods drive an increase in culturable cell levels in water from the tap [2, 16, 18], that can then be reduced through flushing in the morning or after a weekend [8, 9]. However, short stagnations can occur throughout the day, between water usages. To understand the impact of short stagnation on the water microbial quality, a one-hour stagnation following a 5-minute flush was studied and compared to stagnation periods of one to ten days. In cold water, the culturable cell concentrations after flushing were 0.9 x 10^−1^ CFU/mL for tap 1 and 2.7 x 10^−1^ CFU/mL for tap 2. After one hour of stagnation, the tap was sampled and the resulting culturable cell profile was fitted by a power regression (Fig 2A, R^2^ = 0.87), similar to observed profiles after longer periods of stagnation (1 to 10 days). However, culturable bacteria measured in the first 15 mL of water collected after one hour of stagnation were 10- to 100-fold lower than after stagnation periods of 24 hour or more (Fig 2A), suggesting the benefit of flushing in the morning or after the weekend.

The similar results measured in cold water after 24-hour to 10-day stagnation (Fig 2A) raise questions regarding the length of stagnation after which flushing should be performed as a preventive measure to reduce bacterial load. Current guidelines and regulations require flushing at a frequency varying from daily to every other week for low use faucets [8, 9, 34]. However, since bacterial load measured was not systematically higher with increasing stagnation time between 24h and 10-day, investigation should be pursued to confirm the need for daily flushing of infrequently used taps or in sections of buildings that are unoccupied vs flushing prior to usage.

In hot water, the initial culturable cell load measured in the first 15 mL was comparable to levels observed after longer stagnation times: 1.0 x 10^3^ CFU/mL after 1-hour vs a mean value of 3.0 x 10^3^ CFU/mL for all other stagnation times (Fig 2B). Similar to cold water, the increase in culturable cells observed after water stagnation within the tap was predominantly within the first 500 mL of water sampled (Fig 2B).

The increase in culturable cells density in bulk water after a short stagnation can be associated with several phenomena, including: 1) cell growth; 2) regain of culturability; and 3) detachment of culturable cells from the biofilm. Although cell growth is a source of increase reported for prolonged stagnation [16], it is unlikely in the present study due to the short stagnation of one hour and considering the average HPC generation rate in drinking water of 7 to 140 hours [35]. A regain of culturability could occur in the faucet, where the absence of chlorine residual and better availability of oxygen provide favorable conditions [13]. However, documented culturability recovery for drinking water stressed cells occurred over the course of several hours and is unlikely to be significant within a one-hour period [32, 33]. The most likely phenomenon to drive the rapid increase in bulk water culturable cell density after short stagnation is therefore the detachment of bacteria from the biofilm, following two main mechanisms: cell dispersion and biofilm erosion [36]. During periods of stagnation, shear stress is reduced to zero and affects the cell adhesion strength, favoring the release of planktonic bacteria into the water phase [37]. Biofilm erosion or cell sloughing may occur at the end of the stagnation period, marked by an increased flow of water susceptible to erode the biofilm cells that are closest to the bulk water interface [38]. In a previous study, a small increase in flowrate for a short period of time was sufficient to increase cell detachment by 2 log, resuming to initial levels within 3 hours or less after the event [39]. Daughter cells produced at the biofilm interface and cells not embedded in the biofilm matrix are prone to dispersion and erosion mechanisms, and would likely be culturable [40].

Repeated release of culturable cells from the biofilm into the water during stagnation would not significantly affect the biofilm microbial population density despite the slow growth rate reported for HPC in biofilms from drinking water [41]. The fraction of bacteria released from the biofilm is minimal compared to the attached cell counts. In this study, the maximum culturable cells released during one-hour stagnation period in the first 15 mL volume was estimated to 4x10^2^ CFU, a small fraction of the reported biofilm culturable cell densities in premise plumbing, ranging between 2.8 x 10^5^ CFU/cm^2^ and 3.1 x 10^6^ CFU/cm^2^ [3, 42].

**Fig 5:**
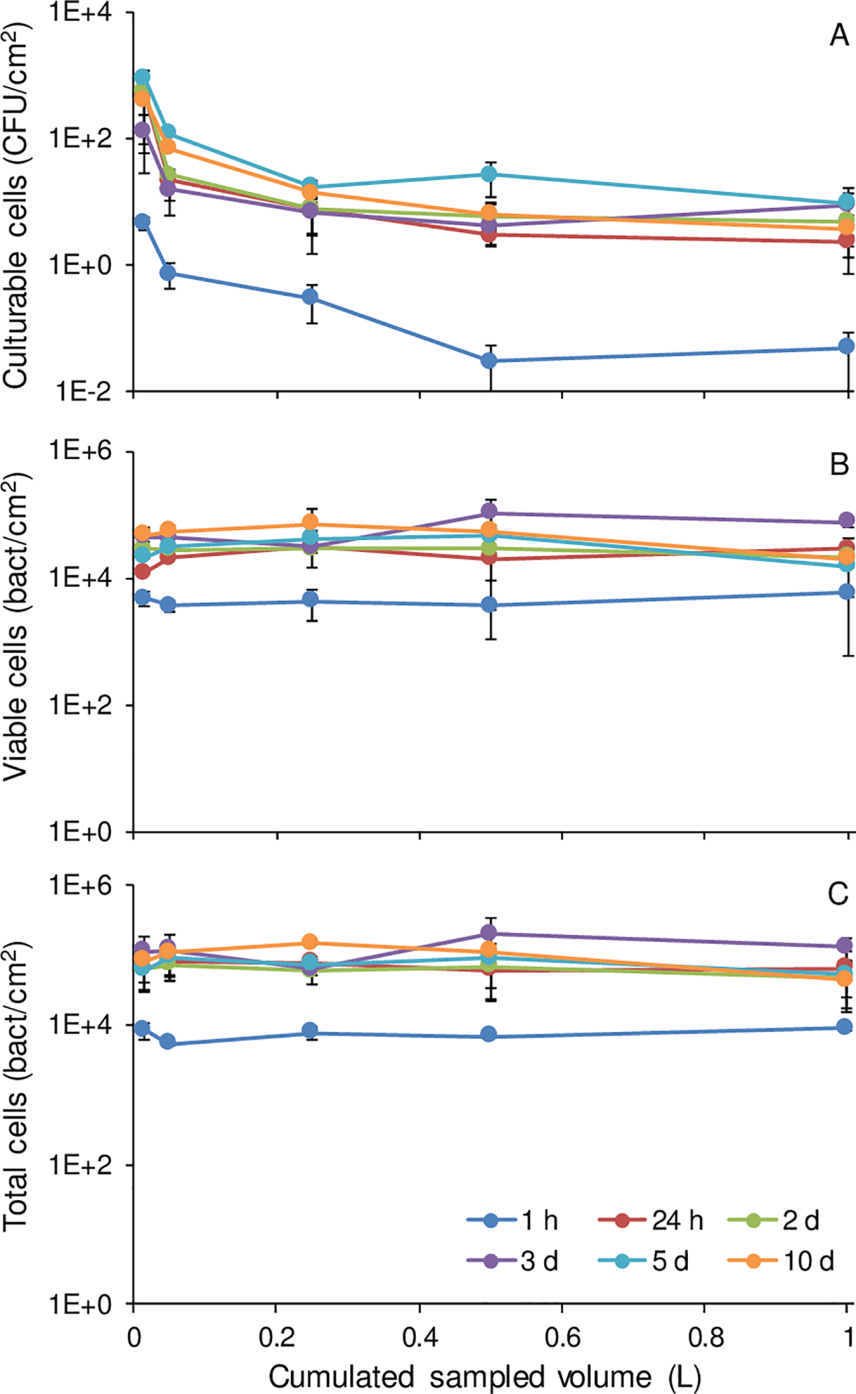
Apparent cell release per unit of pipe surface in cold water during stagnation at the tap. Release of bacteria was calculated based on cell increase over the duration of the stagnation period, for (a) culturable, (b) viable and (c) total cells.

A cell release per unit surface was calculated for each sampled volume using the increment over a defined period of stagnation. In cold water, the apparent culturable cell released per surface was highest in the first 15 mL and the following 35 mL volumes (Fig 5A), corresponding to smaller diameter pipes. Viable and total cell release per surface were not affected by the surface-to-volume ratio, but decreased with increasing stagnation time (Fig 5B and 5C). However, culturable, viable and total cell released within one hour of stagnation were significantly lower than for all other stagnation times. This was not observed in hot water, where a similar number of cells were released per surface for the one-hour stagnation time compared to longer stagnation (S4 Fig). Warmer water temperature and absence of residual chlorine at the beginning of the stagnation could explain in part the different dynamic observed between hot and cold water cell release within the one-hour stagnation period.

### Importance of sampling protocol on results interpretation

**Fig 6:**
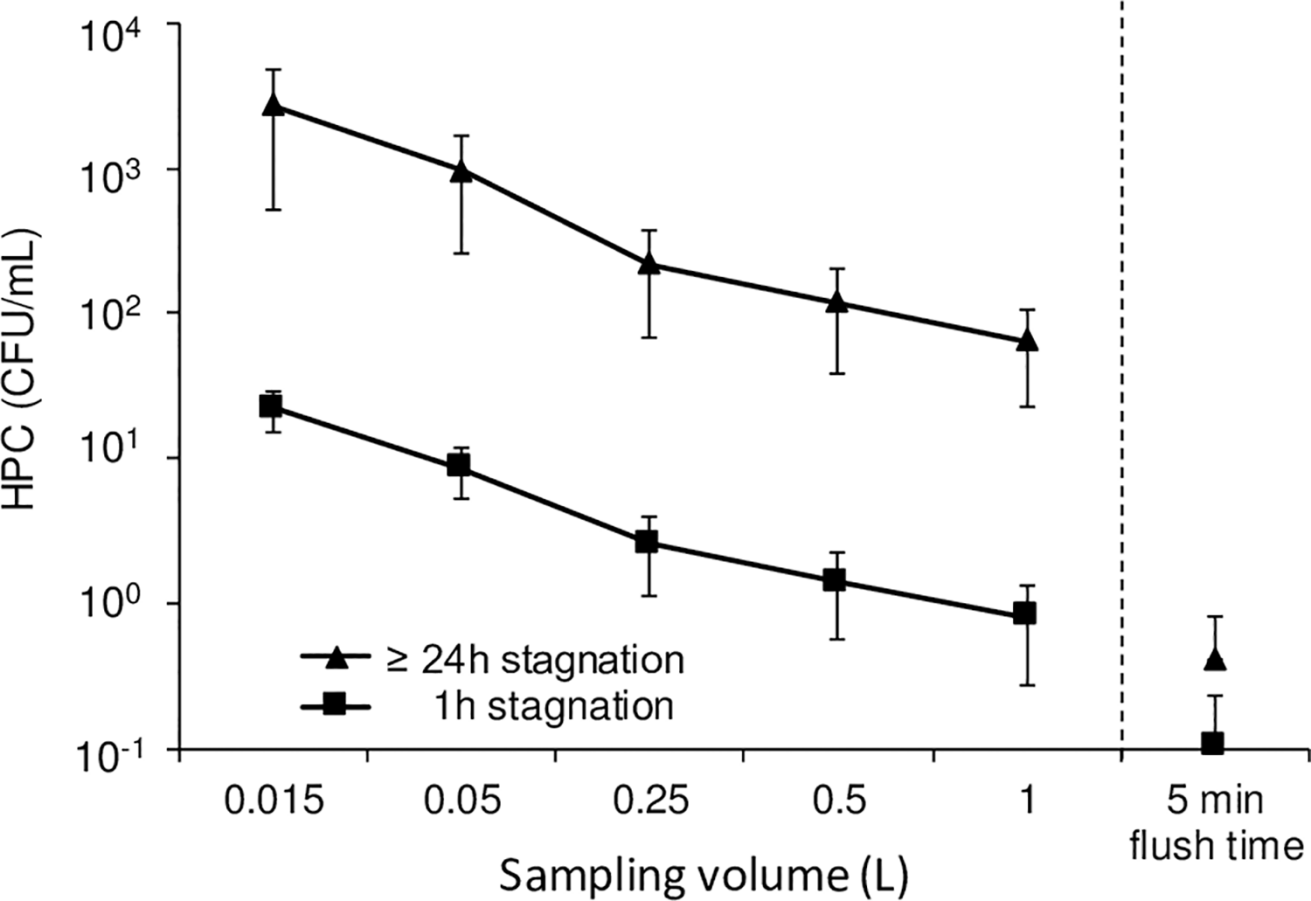
Mean HPC concentration calculated for the cumulated sampling volume after 1 h stagnation (n = 2) and 24h or more of stagnation (n = 10). HPC concentrations is shown as a function of sampling volume for the first liter collected at the tap and after 5 minutes of flush time.

There is currently no consensus on the sampling volumes to use for the detection of indicators and opportunistic pathogens such as *Pseudomonas aeruginosa* and *Legionella pneumophila* [43]. The data presented here demonstrate that the sampling protocol, including sample volume and prior stagnation, will impact the resulting culturable cell concentration measurements. For example, sampling a volume of 15 mL instead of 1 L on first draw led to more than 10-fold higher culturable bacterial levels, whereas sampling after overnight stagnation vs after a short stagnation led to 100-fold increase (Fig 6). Overall, more than 50% of culturable bacteria in the first liter were recovered in the first 15 mL (3.4 x 10^4^ CFU vs 5.5 x 10^4^ CFU). In a previous study, we observed similar trends for *P*. *aeruginosa*, with concentrations in the first 25 mL six to nine times higher than in the first liter, and a decrease in culturable *P*. *aeruginosa* as a function of water volume flushed from the taps [44]. In a healthcare environment, the increased contamination in the distal volume is concerning for opportunistic pathogen control. In such setting, the selection of a small sampling volume on first flush would be preferable to evaluate distal contamination and to increase chances of bacteria recovery. Furthermore, sampling during periods of no or low water use will maximize the recovery of planktonic bacteria.

### Conclusion

The strong correlation observed between the surface-to-volume ratio and the bacteria levels found in the bulk water after one-hour stagnation suggests the biofilm as a major contributor for water contamination following stagnation. Although this correlation needs to be validated for other building systems with different water quality or fed by unchlorinated water, this is an important consideration for healthcare facilities opting for reduced faucet and connecting pipe diameters to achieve flow reduction or to reduce the volume of water that is not recirculated. Such design choices will lead to increased surface-to-volume ratio, and risk of infection should be carefully assessed when considering the implementation of water saving actions and devices. The selection of faucets with minimum internal surface areas, materials with low biofilm formation potential and minimal stagnant water volume in the faucet and its connecting pipe would be a valid approach to reduce available surfaces for biofilm growth and to minimize the bacterial increase associated to stagnation. In case of extended non occupancy of a sector of the building, a daily flush may not present additional benefits compared to a weekly flush, considering the similar bacterial profiles observed in water collected at a tap following stagnation periods between 24-hour and 10 days. However, in presence of low use taps or after stagnation of 24h or more, flushing the volume of stagnant water specific to the tap and its connecting pipes will eliminate a large proportion of the culturable cells. Established bacterial load profiles evidenced the high level of culturable cells present in water from the tap after stagnation periods. Discarding the first flush of water may help reduce exposure to the elevated initial culturable bacterial load observed, especially in hospital environment where patients are more vulnerable to opportunistic pathogens. Sampling protocols in healthcare facilities should take into account the increased distal culturable bacterial load when defining sampling volume. Consideration should be given to systematically include sample volume and prior stagnation in guidelines and regulations for the control of indicators and opportunistic pathogens in premise plumbing. Standardized sampling protocols will enable a better risk assessment over time and better interpretation of results against targeted thresholds for infection prevention.

## Supporting information

S1 Fig Images of a cartridge inside a monolever manual faucet. (TIFF)

S2 Fig HPC concentration as a function of surface-to-volume ratio in cold and hot water at two different taps (Tap 1, Tap 2) for controlled stagnation time of 24h (a), 48h(b), 72h (c), 120h (d) and 240h (e).
(TIF)

S3 Fig Heterotrophic plate counts correlation with total cell counts (full circle) and viable cell counts (empty circle) in tap water at a) first flush volume (15 mL) and b) after 2-minute flush.
(TIF)

S4 Fig Calculated cell detachment rate in hot water from the tap. Cell detachment rate was calculated based on the total cell increase over the duration of the stagnation period, for (a) culturable cells, (b) viable cells and (c) total cells.
(TIF)
