# Supplementary figures and images for: Impact of stagnation and sampling volume on water microbial quality monitoring in large buildings
Source: PLoS One. 2018 Jun 21;13(6):e0199429. doi: 10.1371/journal.pone.0199429 (PMC6013212; doi:10.1371/journal.pone.0199429)

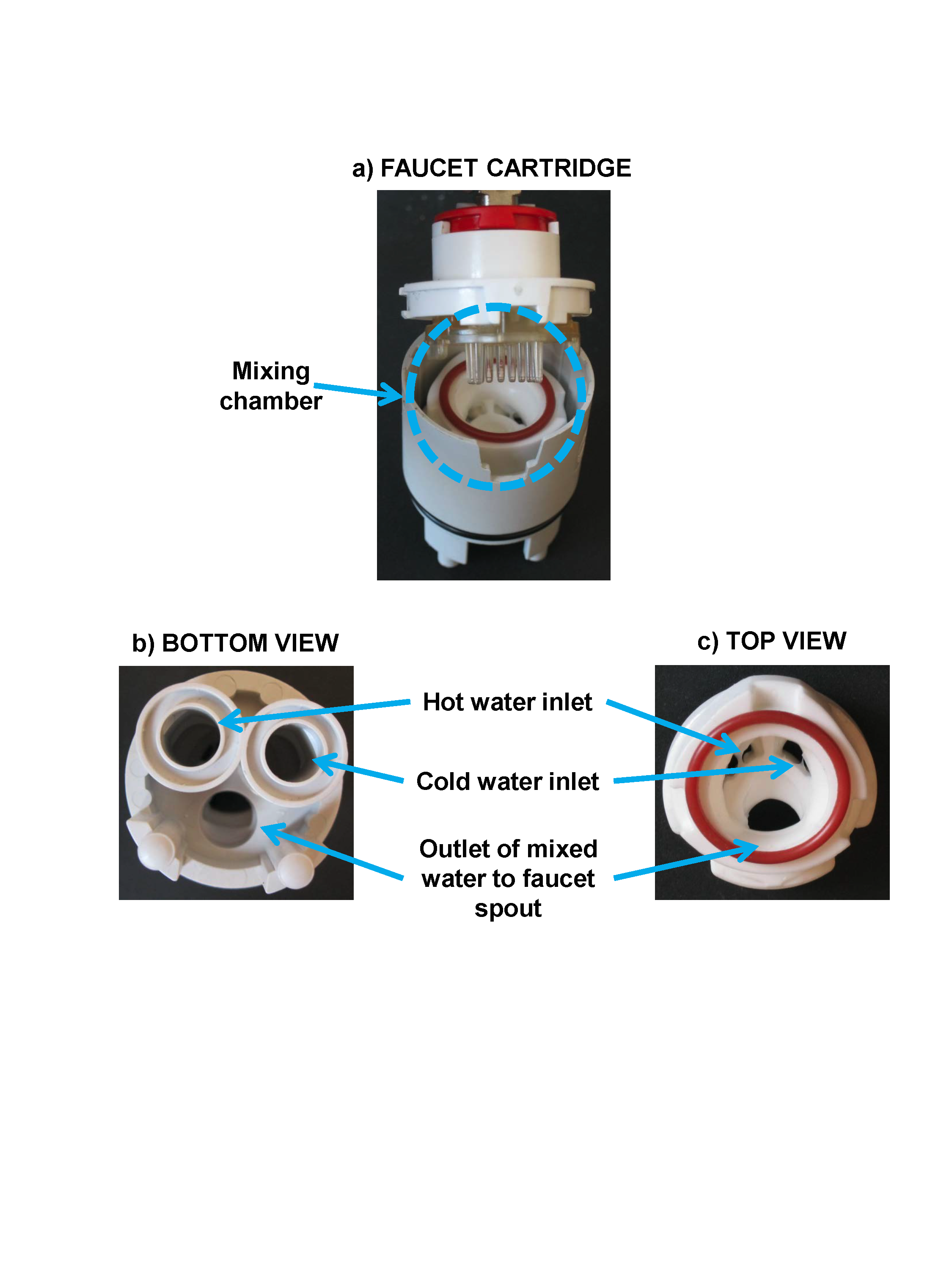

Supplement: S1 Fig — (TIFF) [file pone.0199429.s001.tiff]

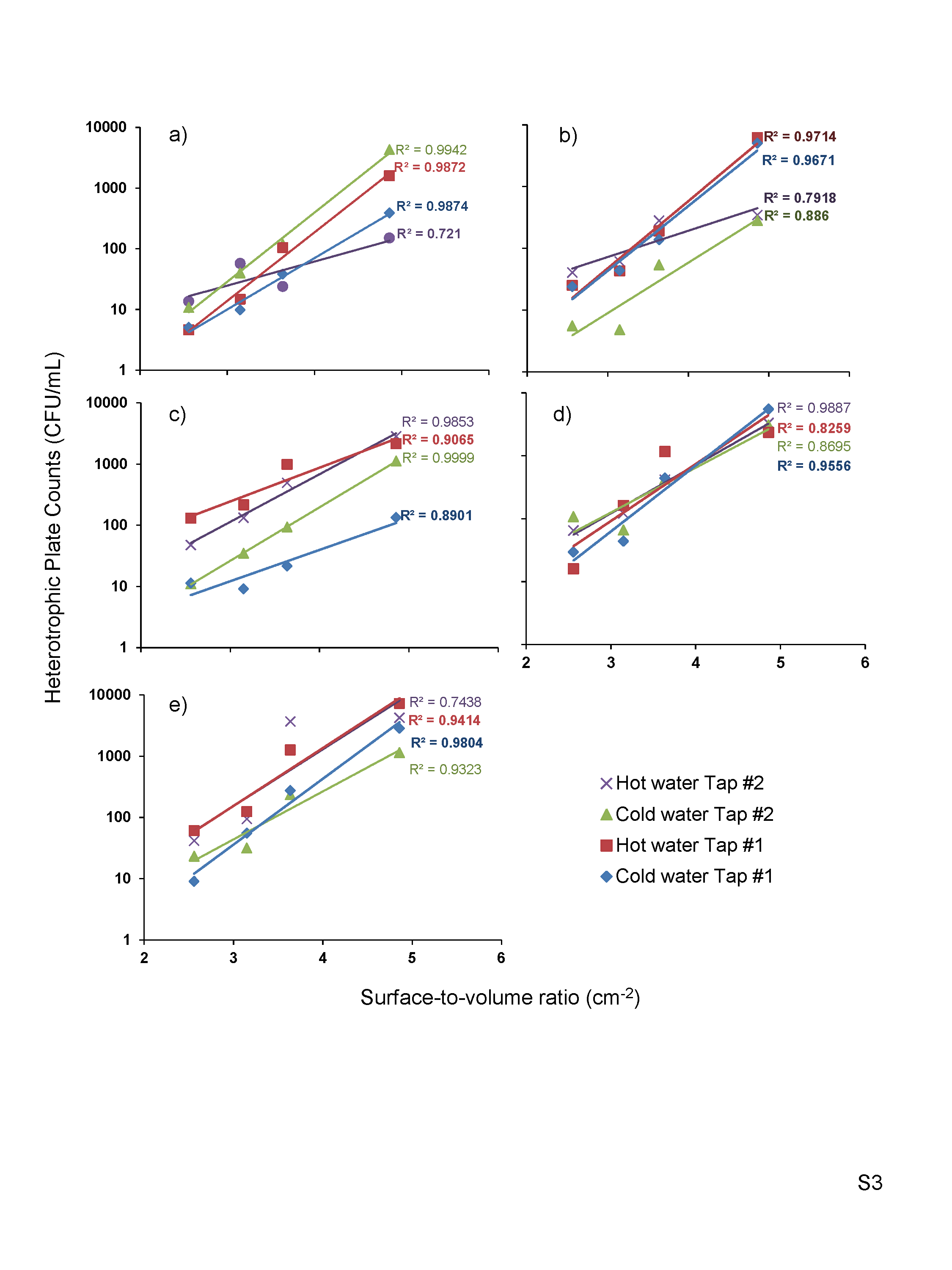

Supplement: S2 Fig — HPC concentration as a function of surface-to-volume ratio in cold and hot water at two different taps (Tap 1, Tap 2) for controlled stagnation time of 24h (a), 48h(b), 72h (c), 120h (d) and 240h (e). (TIF) [file pone.0199429.s002.tif]

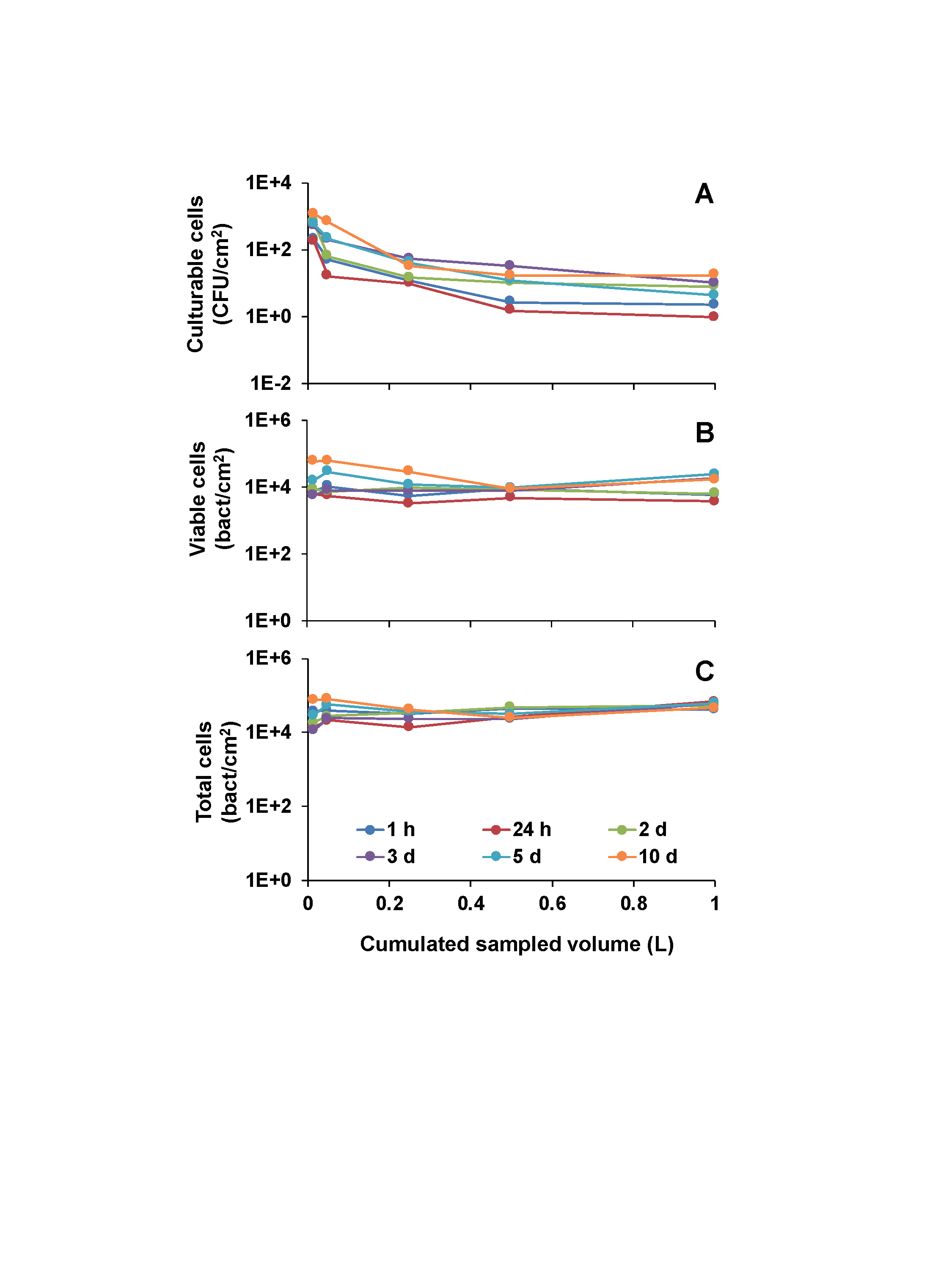

Supplement: S4 Fig — Cell detachment rate was calculated based on the total cell increase over the duration of the stagnation period, for (a) culturable cells, (b) viable cells and (c) total cells. (TIF) [file pone.0199429.s004.tif]
